# Pleural fluid characteristics of patients with COVID‐19 infection

**DOI:** 10.1111/crj.13744

**Published:** 2024-03-26

**Authors:** Ryan A. Denu, Victoria Forth, Majid Shafiq

**Affiliations:** ^1^ Department of Medicine, Brigham and Women's Hospital Harvard Medical School Boston Massachusetts USA; ^2^ Division of Cancer Medicine The University of Texas MD Anderson Cancer Center Houston Texas USA; ^3^ Division of Pulmonary and Critical Care Medicine, Brigham and Women's Hospital Harvard Medical School Boston Massachusetts USA; ^4^ Present address: The University of Texas MD Anderson Cancer Center Houston Texas USA

**Keywords:** COVID‐19, Light's criteria, pleural effusion, pneumonia, SARS‐CoV‐2, thoracentesis

## Abstract

**Introduction:**

Pleural effusions are known to occur in many cases of COVID‐19. Data on typical characteristics of COVID‐19‐associated pleural effusions are limited. The goal of this project was to characterize the pleural fluid from patients with COVID‐19.

**Methods:**

We retrospectively collected electronic medical record data from adults hospitalized at a large metropolitan hospital system with COVID‐19 infection who had a pleural effusion and a thoracentesis performed. We assessed pleural fluid characteristics and applied Light's criteria.

**Results:**

We identified 128 effusions from 106 unique patients; 45.4% of the effusions had fluid/serum protein ratio greater than 0.5, 33.9% had fluid/serum lactate dehydrogenase (LDH) greater than 0.6, and 56.2% had fluid LDH greater than 2/3 of the serum upper limit of normal. Altogether, 68.5% of effusions met at least one of these three characteristics and therefore were exudative by Light's criteria. The white blood cell (WBC) differential was predominantly lymphocytic (mean 42.8%) or neutrophilic (mean 28.7%); monocytes (mean 12.7%) and eosinophils (mean 2.5%) were less common.

**Conclusion:**

We demonstrate that 68.5% of pleural effusions in patients with COVID‐19 infection were exudative and hypothesize that COVID‐19‐associated pleural effusions are likely to be exudative with WBC differential more likely to be predominantly lymphocytic.

## Graphical abstract

The pleural fluid characteristics associated with COVID‐19 infection were assessed in 128 effusions from 106 unique patients; 68.5% of effusions met at least one of the three Light's criteria and therefore were exudative. Elevated LDH was notable. The white blood cell differential was predominantly lymphocytic (in 60.9% of effusions) or neutrophilic (30.5%).

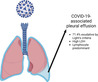

## 1 INTRODUCTION

Severe acute respiratory syndrome coronavirus 2 (SARS‐CoV‐2) is a novel member of the coronavirus family of viruses and is known to cause coronavirus disease 2019 (COVID‐19), responsible for the pandemic that was declared by the World Health Organization in March 2020.^1^ COVID‐19 can cause a number of respiratory manifestations ranging from upper respiratory infection to severe acute respiratory failure.^2^ Typical imaging findings in COVID‐19 pneumonia include bilateral pulmonary parenchymal ground glass and consolidative opacities.^3^ Initial radiographic reports of COVID‐19 pneumonia generally demonstrated lack of pleural effusion.^3^, ^4^ However, accumulating evidence has shown that pleural effusions occur in about 2% to 10% of COVID‐19 pneumonia^5^, ^6^, ^7^, ^8^, ^9^ and up to 23% in patients hospitalized with COVID‐19.^10^ This is comparable to the incidence of pleural effusion in other viral pneumonias, which has been reported to range from 1% to 7%.^11^ The presence of pleural effusion is associated with longer hospital stay and higher mortality.^10^, ^12^, ^13^

This project was catalyzed by a patient we saw with COVID‐19 and pleural effusion as well as other comorbidities that could have explained the effusion. We realized that the pleural fluid characteristics in COVID‐19‐associated effusions have not been well characterized in the literature. In this study, we aimed to better assess the pleural fluid characteristics of patients with COVID‐19 infection.

## 2 METHODS

We queried the Mass General Brigham Research Patient Data Registry (RPDR) electronic health record database for patients with COVID‐19 who had undergone thoracentesis between 1 March 2020 and 1 April 2022. Cases were removed if they did not have at least two fluid chemistries associated with them. We verified that subjects had a positive SARS‐CoV‐2 test ± 2 weeks of the date of the pleural fluid collection. If there were multiple values of a lab associated with the same pleural fluid sample, we took the mean of these multiple values. Cases were excluded if the gram stain or culture were positive. This yielded 128 effusions from 106 unique medical record numbers.

Light's criteria define an exudate by the presence of any one of the following: fluid protein/serum protein > 0.5, fluid lactate dehydrogenase (LDH)/serum LDH > 0.6, and fluid LDH > 2/3 of the serum upper limit of normal (140 U/L).^14^ If lab values exceeded the assay limit, we re‐coded them as the upper limit value.

Two‐tailed *t* tests and one‐way ANOVA were used for comparisons. For multiple comparisons, Tukey's correction was made. *P* values <0.05 were considered significant for all tests, and designations are made in the figures for statistical significance. GraphPad Prism (version 9) was utilized for descriptive statistics and data display.

## 3 RESULTS

**FIGURE 1:**
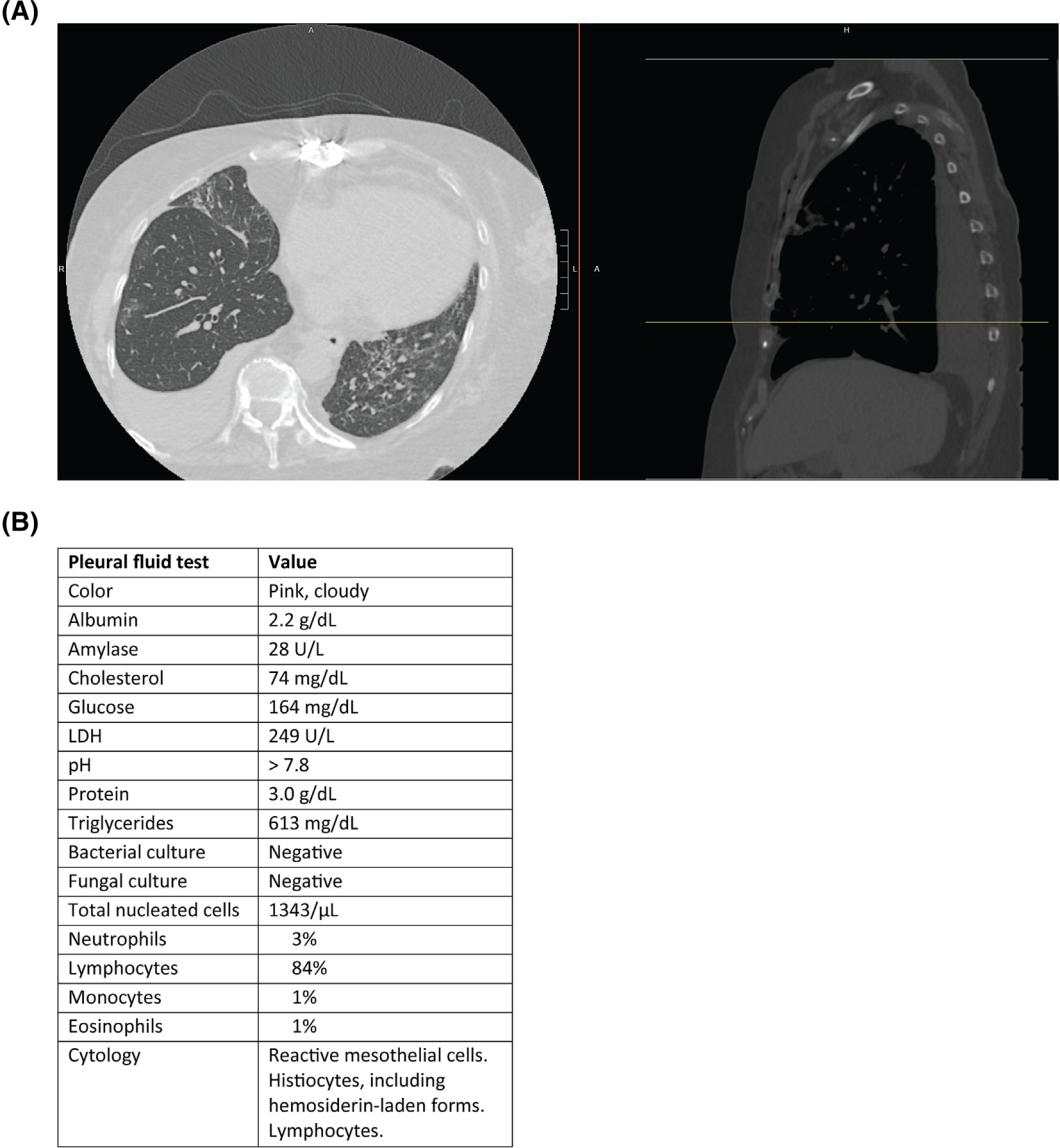
Pleural effusion in patient with COVID‐19. (A) CT chest on admission of 69‐year‐old female patient with recent heart transplant who presented with hypoxia and shortness of breath and tested positive for SARS‐CoV‐2. She was found to have a large right‐sided pleural effusion. (B) Table showing the pleural fluid characteristics, notable for elevated triglycerides.

This project was motivated by a 69‐year‐old female with history of heart transplant 3 months prior and on immunosuppression who presented with cough and shortness of breath, was found to be hypoxic with oxygen saturation of 85% on room air, and tested positive for SARS‐CoV‐2. Computed tomography (CT) of the chest showed a large right‐sided pleural effusion (Figure 1A), which was assumed to be caused by COVID‐19 pneumonia. A thoracentesis was performed, and the pleural fluid had high levels of triglycerides (Figure 1B). We reasoned that this effusion was most likely a chylothorax, though we wondered whether this effusion was related to COVID‐19 infection and aimed to address the dearth of information in the literature regarding COVID‐19‐associated pleural fluid. We queried our health system for pleural fluid characteristics in patients with COVID‐19 who had undergone a thoracentesis (*n* = 128 effusions from 106 unique patients).

**FIGURE 2:**
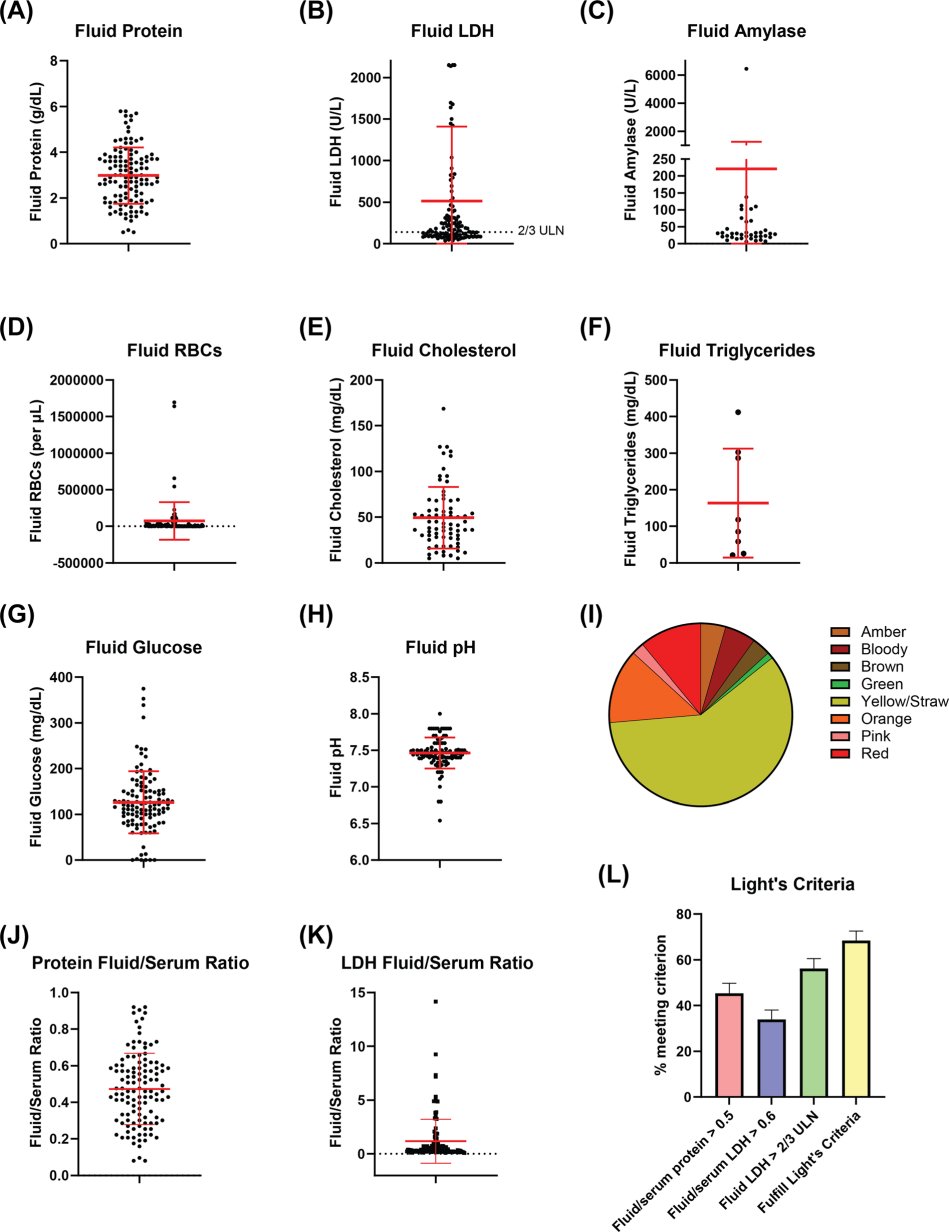
Pleural fluid characteristics in COVID‐19 pneumonia. (A–H) Dot plots showing the distribution of each pleural fluid study. Each dot represents a unique pleural fluid specimen. Red bars represent means ± SD. (A) Fluid protein. (B) Fluid LDH. (C) Fluid amylase. (D) Fluid red blood cells. (E) Fluid cholesterol. (F) Fluid triglycerides. (G) Fluid glucose. (H) Fluid pH. (I) Pie chart demonstrating the distribution of fluid color. (J) Ratio of fluid protein to serum protein. (K) Ratio of fluid LDH to serum LDH. In A‐B, each dot represents a unique pleural fluid specimen. Red bars represent means ± SD. (L) Percentage of effusions that satisfy each of the three individual Light's criteria ± SE of proportion. The final bar on the right indicates the percentage of effusions that satisfy Light's criteria to be classified as an exudate by meeting at least one of the three individual Light's criteria.

COVID‐19‐associated effusions had a mean fluid protein of 3.0 g/dL (range 0.3–5.8, SD 1.23; Figure 2A), mean fluid LDH of 514.4 U/L (range 36–6200, SD 894.8; Figure 2B), mean amylase of 220.9 U/L (range 6–6448, SD 1029.7; Figure 2C), mean red blood cells (RBCs) 73 118 per microliter (range 0–1 695 000, SD 257 406; Figure 2D), mean cholesterol 49.4 mg/dL (range 5–168.5, SD 33.7; Figure 2E), mean triglycerides 163.6 mg/dL (range 21–412, SD 149.0; Figure 2F), mean glucose 126.3 mg/dL (range 0–375, SD 67.8; Figure 2G), and mean pH 7.46 (range 6.54–8.0, SD 0.21; Figure 2H). The most common color was yellow or straw (Figure 2I).

We utilized Light's criteria to characterize the fluid as transudative or exudative. The mean fluid/serum protein ratio was 0.47 (range 0.08–0.92, SD 0.20; Figure 2J), and the mean fluid/serum LDH ratio was 1.17 (range 0.08–14.2, SD 2.04; Figure 2K). Applying Light's criteria, 45.4% of the effusions had fluid/serum protein ratio greater than 0.5, 33.9% had fluid/serum LDH greater than 0.6, and 56.2% had fluid LDH greater than 2/3 of the serum upper limit of normal (Figure 2L). Taken together, 68.5% of effusions met at least one of the three criteria and therefore satisfied Light's criteria for being classified as exudative (Figure 2L).

**FIGURE 3:**
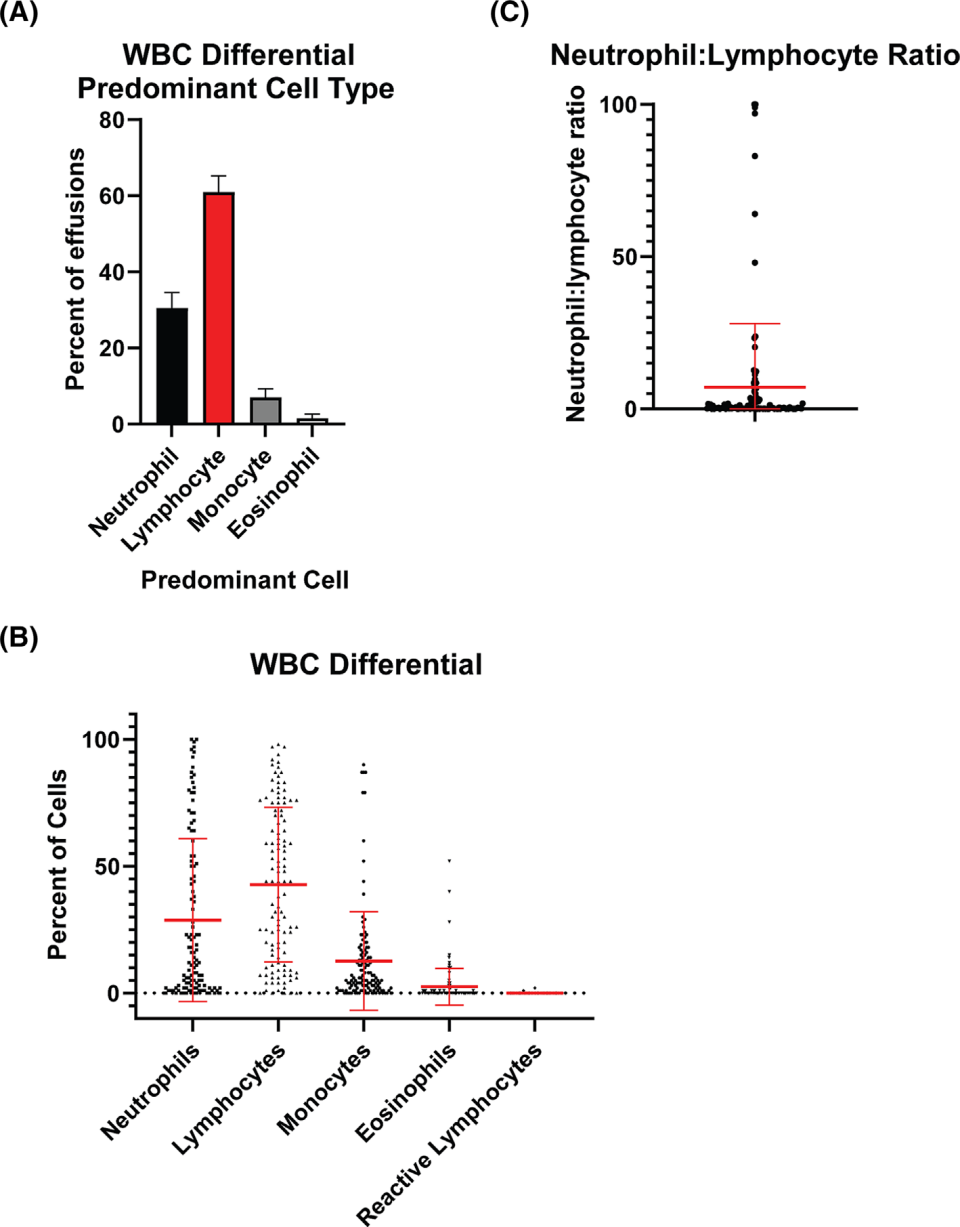
White blood cell differential in COVID‐19‐associated effusions. (A) Percent of effusions with neutrophil, lymphocyte, or monocyte predominance. Bars represent proportions ± standard error of proportion. (B) Distribution of differential for white blood cell subsets in pleural fluid. Each dot represents a unique pleural fluid specimen. (C) Distribution of neutrophil to lymphocyte ratio. In (B) and (C), red bars represent means ± SD.

Regarding fluid white blood cell (WBC) differential, the lymphocyte made up the largest differential in 60.9% of effusions compared with 30.5% neutrophil‐predominant and 7.0% monocyte‐predominant effusions (Figure 3A). The mean neutrophil percentage was 28.7 (range 0–100, SD 32.1). The mean lymphocyte percentage was 42.8 (range 0–98, SD 30.5). Monocytes (12.7% ± 19.4), eosinophils (2.5% ± 7.2), and reactive lymphocytes (0.05% ± 0.28) made up smaller percentages (Figure 3B). The mean neutrophil to lymphocyte ratio was 7.1, and the median was 0.31 (range 0–100, SD 20.9; Figure 3C).

Next, we compared these pleural fluid characteristics to those previously reported in the literature. We identified 10 total cases (Table 1).^15^, ^16^, ^17^, ^18^, ^19^, ^20^, ^21^ Some of these cases were initially compiled into a previously published series,^15^ though we have modified the chart and added additional cases that were published after this review. All 10 pleural effusions were exudative. The mean LDH in these 10 cases (mean 1063 U/L ± 1262, range 79–3651) was higher than our reported LDH (*P* < 0.01), as was the fluid/serum LDH ratio (mean 4.7 ± 7.5, range 0.30–22.8, *P* < 0.01). More effusions were lymphocyte predominant (6/9 = 66.7%, mean lymphocytes 54.3% ± 36.3%) than neutrophil predominant (3/9 = 33.3%, mean neutrophils 34.0% ± 37.1%), and this difference was not significantly different from our series.

**TABLE 1:** Summary of published cases of COVID‐19‐associated effusions.

Age	72	12	68	62	50	50	46	61	71	52
Color	Yellow	Yellow	Serosanguinous	Serous	Serous	Serosanguinous	Sanguinous	Yellow	Serosanguinous	Turbid orange
Cell count (/μL)	120	5860	1815	2719	476	600	7738	25	993	2450
Neutrophils	0	2	75	0	11	47	96			41
Lymphocytes	92	98	9	75	50	30	1		89	45
Eosinophils	0	0	0	0	0	1	1			9
Monocytes	0	0	5	10	39	22	2			
RBCs (per mm^3^)			555 000	88 000	2000	133 000	1 010 000			
pH	7.35		7.45	7.43	7.72	7.8	7.57			7.5
Protein (g/dL)	2.3	4.5	4.5	3.6	2.6	2.2	3.1	2		6
Glucose (mg/dL)	115		132	116	209	191	102			122.52
LDH	168	291	2689	672	549	284	3651	79		1185
Cholesterol (mg/dL)	50	69.61								
Serum LDH	257		904	434	220	220	160	262	363	214
Serum Protein (g/dL)	5.1		4.8	6.3	6.1	6.1	4.9			7.6
Fluid/serum protein	0.45098		0.9	0.6	0.4	0.4	0.6			
Fluid/serum LDH	0.653696		3	1.5	2.5	1.3	22.8	0.301527		5.537383
Exudative?	Yes	Yes	Yes	Yes	Yes	Yes	Yes	Yes	Yes	Yes
Reference	Mei	Chen	Chong	Chong	Chong	Chong	Chong	Bennett	Malik	Hussein

## 4 DISCUSSION

Pleural fluid characteristics can be helpful in making a diagnosis. For example, the case that motivated this project was influenced significantly by the pleural fluid characteristics. Therefore, it can be helpful to know the typical characteristics of pleural fluid in COVID‐19 infection to determine if the pleural effusion is due to COVID‐19 or another cause.

We found that most COVID‐19‐associated effusions are exudative and lymphocyte predominant. Pleural fluid is predominantly produced by the parietal pleural and reabsorbed by the pleural lymphatics, and exudative effusions are thought to arise due to local disease in the pleura and increased capillary permeability. The lymphocyte predominance seen in COVID‐19 infection is similar to what has been reported with other viral pneumonias, whereas neutrophilic predominance is often associated with an acute bacterial pleural process.^11^, ^22^

Prior case reports and series have demonstrated the pleural fluid characteristics in a total of 10 patients,^16^, ^17^, ^18^, ^19^, ^20^, ^21^ as we summarized herein. All were exudative based on Light's criteria; by comparison, 68.5% satisfied Light's criteria in our larger series of 128 effusions from 106 unique patients. The reported white cell count differential in COVID‐19‐associated pleural effusions was either lymphocyte or neutrophil predominant,^6^ consistent with our findings. The LDH elevation in our series was notable, though LDH was even higher in the previously published cases. Some of these differences may reflect more severe cases reported in the literature. Given the small sample size of the previously reported cases (*n* = 10) compared with our series (*n* = 128), there is greater potential for outlier bias, and the values reported in our larger series more likely reflect the distribution of pleural fluid characteristics in COVID‐19.

Our study has several limitations. First, data/studies were not ordered by the clinician or were missing for many of the effusions, limiting analysis. However, nearly all samples had at least fluid protein and LDH available. Second, it is conceivable that some (or many) of the effusions included in the analysis were caused by something other than COVID‐19, such as congestive heart failure or cancer. Given the retrospective nature of this analysis and limited amount of ancillary testing available, it was impossible to fully ascertain the underlying etiology of each effusion. Lastly, the results are limited to hospitalized patients who likely had severe COVID‐19 pneumonia.

In summary, we describe pleural fluid characteristics in COVID‐19‐associated pleural effusions, finding that most effusions are exudative and are characterized by elevated LDH and lymphocyte predominance.

## AUTHOR CONTRIBUTIONS

Ryan A. Denu designed the study, collected data, analyzed data, and wrote and reviewed the paper. Victoria Forth collected data and reviewed the paper. Majid Shafiq designed the study and reviewed the paper.

## CONFLICT OF INTEREST STATEMENT

The authors report no conflicts of interest.

## ETHICS STATEMENT

This project was reviewed and approved by the Brigham and Women's Hospital IRB (protocol 2022P000656, approved 3/31/2022). This was a retrospective study using deidentified data, and the IRB approved a waiver of informed consent.

## Data Availability

The data that support the findings of this study are available from the corresponding author upon reasonable request.
